# A Closer Look at the Birth Order Effect on Early Cognitive and School Readiness Development in Diverse Contexts

**DOI:** 10.3389/fpsyg.2022.871837

**Published:** 2022-06-02

**Authors:** Rufan Luo, Lulu Song, I-Ming Chiu

**Affiliations:** ^1^Department of Psychology, Rutgers University–Camden, Camden, NJ, United States; ^2^Department of Early Childhood Education/Art Education, Brooklyn College, The City University of New York, New York, NY, United States; ^3^Department of Economics, Rutgers University–Camden, Camden, NJ, United States

**Keywords:** birth order, cognitive skills, school readiness, cumulative risks, language minority, diverse context

## Abstract

Using the Early Childhood Longitudinal Study–Birth Cohort data, we examined the effect of birth order (firstborn vs. later-born) on children's cognitive skills at 24 months and school readiness (i.e., math and literacy) skills at age 4 years. Previous research in the U.S. using predominantly English-speaking, low-risk samples suggests that firstborns tend to show better early cognitive and school readiness skills than later-born children. However, results of the current study showed that although there was a firstborn advantage in low-risk or English-speaking families, in high-risk or language minority families, later-born children showed equivalent or even better skills than firstborn children. Our moderated mediation models revealed that children's engagement in home learning activities mediated the relation between birth order and developmental outcomes, and families' cumulative risks and language minority status moderated the mediation pathways. These findings underscore the complex associations between birth order and early development in diverse ecological contexts.

## Introduction

The effect of birth order on early childhood development has attracted extensive research interests in the past decades. While the classic birth order theories (Blake, 1981; Zajonc, 1983) recognize the limitation of family resources and propose a firstborn advantage, the social learning theories highlight the supportive role of older siblings through positive sibling relationships and interactions (Bandura, 1977; Whiteman et al., 2011). Furthermore, family dynamics are subject to contextual influences (Bronfenbrenner and Morris, 2006), calling for a consideration of the diverse family contexts when examining the birth order effect.

In the current study, we used a US, nationally representative dataset, the Early Childhood Longitudinal Study–Birth Cohort (ECLS-B), to examine the effect of birth order on children's cognitive skills during toddlerhood and school readiness (i.e., literacy and math) skills at preschool. These early skills set the foundation for children's long-term academic success (Duncan et al., 2007; Ricciardi et al., 2021). Specifically, we asked whether the effect of birth order was mediated by home learning environment, and whether the birth order effect and the mediation pathways were moderated by two contextual factors, children's exposure to cumulative risks and language minority experiences.

### Classic Birth Order Theories

Classic birth order theories, including the resource dilution theory and the confluence theory, primarily focus on the distribution of family resources. The resource dilution theory posits that a family's human capital (e.g., parental attention, quality time with parents), physical (e.g., books, toys), and financial resources are distributed among siblings (Blake, 1981). Firstborn children have exclusive access to family resources early on, thereby receiving more resources than later-born children. The confluence theory states that the number of children curtail the amount and quality of intellectual resources at home (Zajonc, 1983). Firstborn children tend to experience higher-quality home environment than their later-born siblings, and consequently achieve more advanced developmental outcomes.

### Evidence of the Firstborn Advantage

In line with these theoretical hypotheses, previous studies have revealed a firstborn advantage in early cognitive development. Studies using the National Longitudinal Survey of Youth 1979 (NLSY79) have found that firstborn children outperformed later-born children in cognitive assessments at ages 0–3 years, after controlling for characteristics of the child and family (Heiland, 2009; Lehmann et al., 2018). Similarly, firstborn children have been found to show an advantage in general cognitive development at age 4, measured by combined assessments of early verbal, perceptual-performance, and quantitative skills (Barreto et al., 2017).

Few studies have directly examined the association between birth order and early literacy skills. There is some evidence that firstborn preschoolers showed better reading skills (Lehmann et al., 2018), non-word and sentence repetition, and word reasoning skills (Barreto et al., 2017) than later-born children. Other studies have suggested a firstborn advantage in important precursors of literacy development, including children's vocabulary and grammatical skills (Hoff-Ginsberg, 1998; Berglund et al., 2005; Hoff, 2006) and vocabulary growth rate (Zambrana et al., 2012) during toddlerhood. However, some researchers have argued that the firstborn advantage in vocabulary was observed in maternal report but not in child speech or standardized tests (Bornstein et al., 2004).

In terms of math skills, some studies have documented a firstborn advantage in children's math skills during preschool years (Barreto et al., 2017; Lehmann et al., 2018). Yet, there is evidence that the effect of birth order might be weaker for math and non-verbal skills than for verbal cognitive and literacy skills (Pavan, 2016; Peyre et al., 2016; Lehmann et al., 2018). Researchers have argued that some mathematical skills are more likely to be learned at school rather than at home, which might make math skills less susceptible to the influences of birth order (Lehmann et al., 2018). Together, these findings suggest a nuanced relation between birth order and early development, highlighting the importance of understanding the mechanisms behind the birth order effect.

### The Mediating Role of Home Learning Environment

According to the birth order theories, one potential mechanism through which birth order is associated with early childhood development is the home learning environment, which is often measured as children's engagement in learning activities at home (e.g., book-reading, storytelling, etc. Tamis-LeMonda et al., 2019). Firstborn and later-born children may experience different learning environment at home, which may in turn result in disparities in their developmental outcomes.

Some previous studies have shown that being firstborn is positively associated with the quantity and quality of child-directed speech from parents (Hoff, 2006), cognitive stimulation (Peyre et al., 2016), the frequency of shared reading (Raikes et al., 2006), and the amount of quality time spent with parents (Price, 2008). Similarly, using the ECLS-B dataset, Workman (2017) found that the addition of a new sibling was negatively associated with changes in the frequency of learning activities (e.g., storytelling) from 9 to 24 months. There is some, albeit limited, evidence of the mediating effect of home learning environment. In one study, parental use of cognitive and linguistic stimulations and the quality of parent-child interactions when children were 2 years old partially mediated the effect of birth order on children's cognitive skills at age 4 years (Barreto et al., 2017). Another study suggested that, parents spent less time teaching and reading to their later-born children, engaged in learning activities less frequently with their later-born children, and provided fewer age-appropriate toys at home for later-borns (Lehmann et al., 2018). These differences in home learning environment fully explained the firstborn advantage on cognitive skills at ages 0–3 years (Lehmann et al., 2018).

### Challenges to the Classic Birth Order Theories and Firstborn Advantage

Unlike the classic birth order theories, which view siblings as competitors for family resources, the social learning theories consider siblings as socializers and role models who enrich children's learning experiences via positive sibling relationships and sibling interactions such as play and teaching (Bandura, 1977; Whiteman et al., 2011). Siblings learn from one another through observing, imitating, and responding to others' behaviors, attitudes, and beliefs (Whiteman et al., 2011). Particularly, when older siblings elicit unique learning experiences that are not typically offered by parents, later-born children may show an advantage in the specific developmental domain. For example, later-born children have been found to acquire personal pronouns at an earlier age (Oshima-Takane et al., 1996) and have better conversational skills (Hoff-Ginsberg, 1998) than firstborns, probably because they have more opportunities to engage in triadic interactions with their mother and older sibling. Research has also suggested a later-born advantage in developmental domains such as social cognition, emotion regulation, and behavioral adjustment (Hou et al., 2020; Hjern et al., 2021). Sibling interactions may present unique social challenges and learning opportunities (e.g., conflicts solving, sharing, perspective taking), compared to parent-child interactions.

Additionally, the bioecological model suggests that families constantly adapt to the socioecological context they live in and respond to the changing needs and development of each family members (Bronfenbrenner and Morris, 2006). Accordingly, the behaviors and responsibilities of older siblings may vary across families of diverse contexts. For instance, in families of low socioeconomic or immigration backgrounds where parents experience financial hardship or language barriers, older siblings may take more responsibilities in teaching later-born children the host language, engaging them in learning activities, and bridging the home and school cultures (Hurtado-Ortiz and Gauvain, 2007; Obied, 2009; Farver et al., 2013). As a result, instead of suffering from reduced human capital resources, later-born children in these families may benefit from having older siblings who help to enrich their learning experiences in ways their parents cannot.

Together, these theories suggest that having older siblings may be more beneficial in certain contexts than in others, challenging the generalizability of the firstborn advantage. Indeed, a growing body of research in developing, non-Western countries have found evidence of non-significant birth order effects or later-born advantages [e.g., Ejrnæs and Pörtner, 2004; Tenikue and Verheyden, 2010; Seid and Gurmu, 2015; Botzet et al., 2021]. Yet, work in this area has largely focused on intelligence or educational outcomes in adolescence and adulthood. It remains unclear whether the birth order effect on early cognitive and school readiness skills varies across diverse contexts, and even less is known about which contextual factors moderate the effect of birth order and how.

### The Moderating Role of Cumulative Risks and Language Minority Experiences

Here we focused on two contextual factors as potential moderators of the birth order effect on early cognitive and school readiness development. The first one, cumulative risks, referring to the co-occurrence of family risk factors such as poverty, low parental education, single parenthood, and maternal depression, has been found to negatively predict early cognitive and school readiness skills (Burchinal et al., 2000; Stanton-Chapman et al., 2004; Pratt et al., 2016). The second factor is language minority experiences, which may include the use of a minority language at home and parental limited English proficiency and foreign-born status. On the one hand, early exposure to a minority language presents children with the opportunity to become proficient bilinguals and reap the cognitive and social benefits of bilingualism (Bialystok, 2009). On the other hand, language minority families in the U.S. often possess some of the family risk factors such as poverty and low parental education. Therefore, as a group, preschool children from language minority families tend to fall behind their monolingual peers in the host language and school readiness skills (see Hoff, 2018). Although the two contextual factors may have similar effects on early cognitive and school readiness skills, they each capture distinct characteristics of diverse family contexts.

#### Contextual Factors Moderating the Birth Order Effect on Home Learning Environment

Beyond their direct effects on child development, these contextual factors may moderate the effect of birth order on children's home learning environment. Compared to their peers, children from high-risk or language minority families tend to have fewer opportunities to engage in high-quality learning activities, due to financial hardship, limited human capital, and/or language barriers (Raikes et al., 2006; Rodriguez and Tamis-LeMonda, 2011). In these families, where there is likely a disconnection between the home and school environments, the presence of older siblings may enhance, rather than undermine, children' home learning experiences (Hurtado-Ortiz and Gauvain, 2007; Obied, 2009). For instance, low-income mothers reported that their older children's school experiences allowed them to be more aware of the importance of early literacy development (Sawyer et al., 2018), which may lead them to engage the younger siblings in literacy activities more frequently than they did with the firstborn children. In language minority families in the United States, the presence of school-age older siblings positively predicted younger toddler siblings' English skills (Bridges and Hoff, 2014; Hoff et al., 2014), partially because mothers increased their English use at home after their firstborn entered school.

Older siblings in high-risk or language minority families may also take more responsibilities scaffolding their younger siblings' learning than their counterparts would have in other families (Gregory, 2001; see Zentella, 2005). In a qualitative study with Latino immigrant families where parents had limited English skills, Kibler et al. (2016) documented how older siblings supported their younger siblings' bilingual and school readiness skills, by sharing word knowledge and engaging their younger siblings in shared narratives, reading, and writing activities. The contributions of older siblings can be especially valuable when parents offer insufficient language and literacy support due to their language barriers, lack of resources, or other risk factors (Kibler et al., 2016). A study of low-income, ethnic minority families suggested that children with parents who had lower education levels engaged in language and literacy activities more frequently with other family members such as older siblings, than those children with parents who had higher education levels (Tamis-LeMonda et al., 2019).

#### Contextual Factors Moderating the Birth Order Effect on Developmental Outcomes

Given that cumulative risks and language minority experiences may reduce or even reverse the firstborn advantage in children's home learning experiences, these contextual factors may also moderate the effect of birth order on early development. For example, Kim et al. (2018) found that the effect of birth older on Korean children's expressive vocabulary growth from ages 3 to 7 years was moderated by family income, such that there was a firstborn advantage in high-income families but a later-born advantage in low-income families. Likewise, another study with Norwegian toddlers suggested a moderating effect of maternal education on the association between birth order and children's language skills (Zambrana et al., 2012). However, these studies did not consider other risk factors such as maternal depression or single parenthood. Although no study to our knowledge has examined the moderating role of language minority experiences, the firstborn advantage has been found to be small or non-significant in ethnic minority families. Lehmann et al. (2018) found that the firstborn advantages on home learning environment and early cognitive outcomes were only observed in White families but not in ethnic minority families.

### The Current Study

Previous studies have demonstrated an association between birth order and early developmental outcomes. Yet, the underlying mechanisms of the birth order effect still need to be explored. More importantly, little is known about whether the birth order effect generalizes across diverse contexts and if not, which contextual factors moderate the direction or magnitude of the effect. Answers to these questions will provide a more accurate picture of the role of birth order in early childhood development and reveal the complex interrelationships among birth order, home environment, and broader ecological context. Findings will also shed light on the unique learning opportunities for children from diverse backgrounds. Specifically, we asked two sets of research questions.

(1a) Does birth order (i.e., firstborn vs. later-born) predict children's cognitive skills at 24 months and school readiness (i.e., literacy and math) skills at preschool (48 months)?

**Figure 1:**
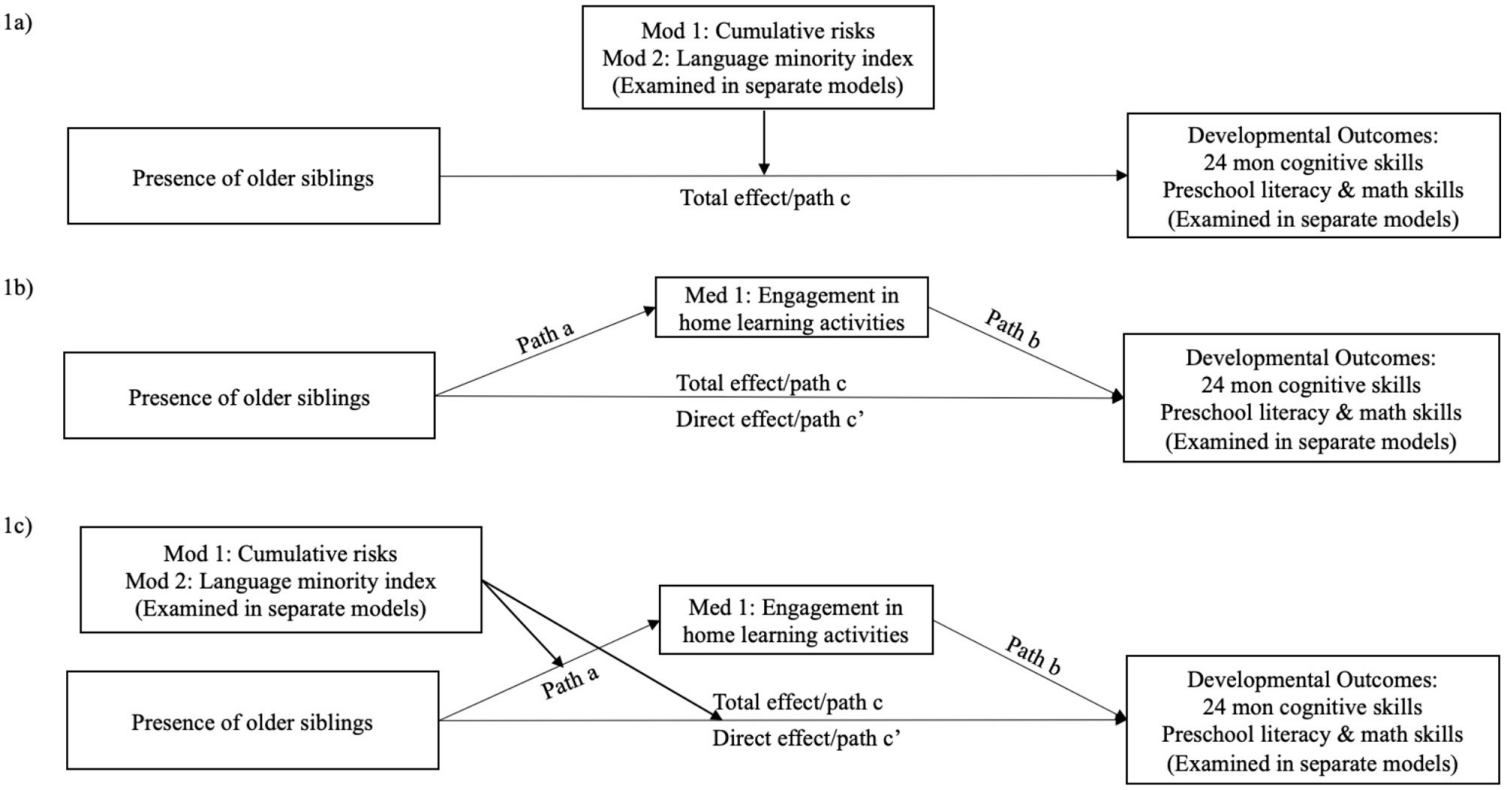
Theoretical frameworks for research questions. **(a)** presents the proposed moderation model for Research Question 1b, in which birth order served as the key predictor, and cumulative risks and language minority risks served as the moderators in separate models. Children's cognitive skills at 24 months and school readiness skills at preschool were the dependent variables in separate models. **(b)** presents the proposed mediation models for Research Question 2a, in which birth order served as the key predictor. Children's engagement in home learning activities was the mediator. Children's cognitive skills at 24 months and school readiness skills at preschool were the dependent variables in separate models. **(c)** presents the proposed moderated mediation models for Research Question 2b, in which cumulative risks or language minority index served as the moderators in separate models. The models tested whether these two variables moderated the pathways from birth order to children's engagement in learning activities and cognitive and school readiness outcomes.

Across the entire sample of diverse families, we expected firstborn children to show more advanced developmental outcomes than later-born children.

(1b) Is the effect of birth order moderated by children's exposure to cumulative risks and language minority experiences at home (see Figure 1A)?

We expected to observe a smaller firstborn advantage or even a later-born advantage in high-risk or language minority families than in low-risk or English-speaking families.

(2a) Does children's home learning environment, as measured by their engagement in learning activities with family members, mediate the association between birth order and child outcomes (see Figure 1B)?

Across the entire sample, we expected being later-born to be negatively related to children's engagement in home learning activities, which in turn is positively related to child outcomes.

(2b) Does the mediation pathway via children's engagement in home learning activities vary by children's exposure to cumulative risks and language minority experiences at home (see Figure 1C)?

We expected both the direct effect of being later-born (i.e., path c') and the indirect effect via home learning activities (i.e., path a) to be less negative or even positive in high-risk or language minority families than in low-risk or English-speaking families.

## Method

### Participants

Data were drawn from the Early Childhood Longitudinal Study–Birth Cohort (ECLS-B), which involved a nationally representative sample of ~10,700 children born in the United States in 2001. The sample was selected from over 14,000 registered births from the National Center for Health Statistics' vital statistics system (see Bethel et al., 2005). Children who died or were adopted before 9 months or born to mothers <15 years of age were excluded from the sample. ECLS-B oversampled children from certain racial/ethnic backgrounds (e.g., Asian and Pacific-Islander, Indian and Alaska Native), twins, and children with low or very low birth weight. At baseline (9 months), 51% of children were male, and 83.6% were singletons. In terms of children's race, 41.5% were non-Hispanic White, 15.9% were non-Hispanic African American, 20.6% were Hispanic, 11.3% were Asian, 2.8% were American Indian/Alaska Native, 0.45% were Native Hawaiian/other Pacific Islander, and the rest were non-Hispanic multiple races. Most children (73.7%) had normal birth weight, and 15.5 and 10.9% of them had moderately low or very low birth weight. Mothers were on average 27.5 years of age (*SD* = 6.36, *range* = 15–50) at the birth of the target child, and 50.2% of them were working at baseline. Table 1 presented demographic information on the families' socioeconomic and linguistic backgrounds. For more information about the study sample, please visit: https://nces.ed.gov/ecls/birth.asp.

**Table 1:** Descriptive statistics for key predictors, mediators, moderators, and outcome variables.

	**24 months**				**Preschool**			
	**Mean/%**	**(SD)**	**Range**	**Missing**	**Mean/%**	**(SD)**	**Range**	**Missing**
Later-born status	56.4%			0.0%	55.7%			0.0%
Cumulative risks index	0.91	(0.99)	0–3	0.6%	0.90	(1.02)	0–3	6.9%
Poverty	24.6%			0.0%	24.4%			0.0%
Father residency	79.1%			0.0%	79.1%			0.0%
Maternal education				0.5%				1.0%
High school or lower	45.0%				39.6%			
Maternal depression^a^	0.68%			0.1%	5.41	(5.74)	0–36	6.0%
Language minority index	0.50	(0.94)	0–3	2.5%	0.48	(0.92)	0–3	2.4%
English as home primary language	79.0%			0.0%	79.8%			0.0%
Mothers' English proficiency^b^	14.94	(2.81)	4–16	2.5%	14.98	(2.75)	4–16	2.4%
Mother foreign born	24.6%			0.0%	23.86%			0.1%
Home learning environment								
Engagement in learning activities^c^	9.31	(2.03)	3–12	0.0%	8.96	(1.98)	3–12	0.0%
Child outcomes								
Cognitive skills	125.53	(10.99)	92.35–174.14	9.3%				
Literacy skills					25.46	(10.50)	11.65–80.29	6.9%
Math skills					29.36	(10.01)	9.83–65.74	7.2%

a *Maternal depression was assessed differently at 24 and 48 months. At 24 months, about 0.68% of mothers reported that they were taking anti-depression medication. At 48 months, mothers reported their feelings of depression using the Center for Epidemiologic Study Depression Scale (CES-D; Radloff, 1977), and their average score was 5.41 on a scale of 0–36*.

b *Mothers' English proficiency was measured by 4 self-reported items about how well the mother was able to understand, speak, read, and write in English (1-not well at all, 4-very well). Higher scores indicated higher levels of English proficiency*.

c *The sum score of three items asking about how often mothers or other family members engaged in book-reading, storytelling, and singing with the target child in a typical week (1-not at all, 2-once or twice, 3-three to six times, 4-every day)*.

*Source: U.S. Department of Education, National Center for Education Statistics, Early Childhood Longitudinal Study, Birth Cohort (ECLS-B), 24 month and preschool data*.

### Procedure

The longitudinal data collection took place when children were on average 9-month-old (*N* = 10,700), 24-month-old (*N* = 9,850), 48-month-old (i.e., preschool wave; *N* = 8,950), and when children first enrolled in kindergarten (*N* = 7,000 in 2006, *N* = 1,900 in 2007). The current study focused on the 24-month and preschool waves, because children were more likely to spend a significant amount of time at home and interacting with their siblings during toddlerhood and preschool years. Each wave of data collection was conducted via a home visit, which included an extensive interview with the child's primary caregiver, typically the mother, observations of parent-child interactions, and direct child assessments.

### Measurements

#### Birth Order

At each wave, parents were interviewed about individuals living in the household. A variable indicating the child's birth order (0-firstborn, 1-later-born) was generated at each wave. We compared children with and without older sibling(s), rather than firstborn and later-born children within the same household. The sample consists of one child per family and children can be either firstborn or later-born. Twin or higher-order multiple births siblings were not counted as older siblings, given the minimal age difference between them and the target child.

#### Cognitive Skills at 24 Months

Children's cognitive skills at 24 months were assessed by a trained administrator using the mental scale of Bayley Short Form–Research Edition (BSF-R), adapted from the Bayley Scales of Infant Development: Second Edition (BSID-II; Bayley, 1993). The mental scale consisted of 33 items designed to assess 2-year-olds' early cognitive and language skills, including memory, expressive and receptive vocabulary, reasoning and problem solving, and concept attainment (Andreassen and Fletcher, 2007). For families where English was not the primary language, the assessment was conducted in the home language either by a bilingual interviewer or via the assistance of an interpreter. In the ECLS-B dataset, scale scores were calculated using the Item Response Theory (IRT) methods. All children received a set of routing items. Based on their performance, children were then routed to a set of supplementary items with low-, middle-, or high-difficulty level. This approach allows an accurate assessment of children's abilities at various levels and minimizes the floor and ceiling effects. The IRT scale scores were generated to estimate the number of items children would have answered correctly if they had received the full set of BSID-II mental scale items (possible range of 0–178). The reliability of the IRT-based scores was 0.94 (Andreassen and Fletcher, 2007).

#### School Readiness (Literacy and Math) Skills at Preschool

Children's school readiness skills, including their early literacy and math skills, were assessed when children were 4 years of age. The literacy assessment consisted of 37 items assessing children's phonological awareness, letter sound knowledge, letter recognition, print conventions, and word recognition skills (Snow et al., 2007). The math assessment included 44 items focusing on number sense, counting, operations, geometry, pattern understanding, and measurement (Snow et al., 2007). Prior to these assessments, children's understanding of English was examined via an English fluency screening test and the parent interview. Children who did not pass the English screening were either routed to the Spanish version of the literacy and math assessments or did not receive these assessments if they spoke a language other than Spanish and English. Given the small proportion of children (1.37%) who received the assessments in Spanish, only data from the English-assessed children were analyzed. The IRT scores (possible range, 0–37 for literacy and 0–44 for math) were generated for children's early literacy and math skills, with reliabilities of 0.81 and 0.88, respectively.

#### Home Learning Environment at 24 Months and Preschool

At the 24-month and preschool waves, children's home learning environment was measured by children's engagement in learning activities. Mothers were interviewed about how often they or other family members engaged in book-reading, storytelling, and singing with the target child in a typical week (1-not at all, 2-once or twice, 3-three to six times, 4-every day). A total score was calculated, ranging between 3 and 12.

#### Cumulative Risks

At the 24-month and preschool waves, the accumulation of multiple risk factors was measured via parent interview. The families received 1 point for the presence of each of the following four risk factors: (1) *Poverty*, indicated by whether or not the annual income of the family was below the federal poverty line in the year of data collection (0-no/no risk, 1-yes/at risk); (2) *Single-mother household*, indicated by whether the father was living in the household (0-yes/no risk, 1-no/at risk); (3) *Low maternal education*, indicated by whether mothers had high school or lower level of education (0-higher than high school/no risk, 1- high school or lower/at risk); and (4) *Maternal depression*. At the 24-month wave, mothers reported on whether they were taking anti-depression medicine (0-no medicine/no risk, 1-taking medicine/at risk). At the preschool wave, a 12-item, abbreviated Center for Epidemiologic Study Depression Scale (CES-D, Radloff, 1977) was used to measure mothers' feeling of depression. A CES-D score of 15 or higher indicated severe depression (0-no severe depression/no risk, 1-severe depression/at risk).

A sum score was calculated to indicate the total number of risk factors the family experienced, with higher scores indicating greater levels of cumulative risks. Because only a small percentage (0.03% at 24 months and 1.27% at preschool) of families had all 4 risk factors, we combined those families with 3 or 4 risk factors into one category. Therefore, the cumulative risks variable ranged from 0 (no risk factor) to 3 (3 or more risk factors).

#### Language Minority Index

At the 24-month and preschool waves, a language minority index was generated based on parent interview to indicate the language minority environment at home. The families received either a 1 or a 0 depending on whether they met the criterion of language minority for each of the following three factors. (1) *Home primary language*. At each wave, a family received 0 if the mother reported English as the primary language used at home. A family received 1 if English was not the primary home language. (2) *Mothers' English proficiency*, measured at baseline (i.e., 9-month wave) by 4 self-reported items about how well the mother was able to understand, speak, read, and write in English (1-not well at all, 4-very well). A family received 1 if the sum score of these 4 items was 8 or below (i.e., relatively low English proficiency) and received 0 if the sum was 9 or above. (3) *Mothers' foreign-born status* was measured at baseline (i.e., 9-month wave). A family received 1 if the mother was born outside of the United States and received 0 if the mother was native born. The language minority index was the sum score of the three factors, ranging from 0 to 3, calculated at each wave; the higher the index, the more likely a minority language was used at home.

#### Covariates

An extensive group of demographic variables were included as covariates in the analyses, including children's sex, age of assessment at each wave, birth weight (i.e., normal, moderately low, very low), multiple birth status (i.e., singleton, twin, higher order), race (non-Hispanic White, non-Hispanic Black or African American, Hispanic, non-Hispanic Asian, Native Hawaiian/other Pacific Islander, American Indian/Alaska Native, non-Hispanic multiple races), mothers' age at child birth, number of siblings at each wave, total number of household members at each wave, mothers' working status (1-yes, 0-no) at each wave, region of residence (i.e., Northeast, Midwest, South, West) at each wave, degree of urbanicity (i.e., urban, suburban, rural) at each wave, the number of hours per week children spent in non-parent childcare arrangement, and mothers' self-reported health (“In general, would you say your health is?” 1-poor, 2-fair, 3-good, 4-very good, 5-excellent). All covariates were associated with at least one of the key variables (i.e., child outcomes and the mediator).

### Analytic Plan

Analyses were performed in Stata 16.1. Wave-specific sampling weights were used in analyses to account for oversampling and attrition and generalize findings to American children born in 2001.The full information maximum likelihood (FIML) estimation method, which allows the use of all available data, was used to handle missing data. As shown in Table 1, the missing rates of key variables were fairly small, ranging from 0 to 9.3%. Compared to parental interviews, there were relatively more missing values in child assessments at age 2, probably due to the challenges of directly assessing children at a young age.

To examine Research Question 1a, a set of multiple regression models were conducted to test the associations between birth order and children's cognitive and school readiness outcomes. We used birth order as the key predictor and children's concurrent developmental outcomes (i.e., cognitive skills at 24 months or literacy and math skills at preschool) as the dependent variables, respectively. The cumulative risk variable, language minority index, and demographic covariates were controlled in these models.

To answer Research Question 1b, whether the effect of birth order on child outcomes varied by cumulative risks and language minority index (see Figure 1A), we tested the moderation effect of cumulative risks in a set of models with the interaction term of birth order × cumulative risks. Significant results of the interaction term would indicate a moderation effect of cumulative risks. We further used Stata's postestimation command, nlcom, to estimate the conditional effects of birth order, when cumulative risks was set at 0 (low level; hereafter referred to as *low-risk families*) or 3 (high level; hereafter referred to as *high-risk families*). We used Chi-square tests to examine whether the conditional effects of birth order significantly differed between low-risk and high-risk families. To reduce multicollinearity, the second moderator, language minority index, was tested in a set of similar, yet separate models. We estimated the conditional effects, by setting language minority index at 0 (low level; hereafter referred to as *English-speaking families*) or 3 (high level; hereafter referred to as *language minority families*). Demographic variables, cumulative risks, and language minority index were controlled in both sets of moderation models.

To examine Research Question 2a, we asked whether children's engagement in home learning activities mediated the associations between birth order and cognitive and school readiness outcomes (see Figure 1B). We used Stata's postestimation command, nlcom, to estimate the indirect and direct effects of birth order. Control variables were the same as in the analyses for the first set of research questions.

To examine Research Question 2b, we evaluated the moderated mediation models to understand the extent to which the direct and indirect effects of birth order on child outcomes varied by cumulative risks and language minority index. As shown in Figure 1C, we examined whether cumulative risks or language minority index moderated two potential pathways: (1) birth order → engagement in home learning activities (path a), and (2) the direct effect of birth order on cognitive or school readiness outcomes after controlling for home learning activities (path c'). To examine the moderation of cumulative risks, the interaction term of birth order × cumulative risks was added to the mediation models described above. The second moderator, language minority index, was tested in separate models. Where there were significant interactions, conditional direct and indirect effects were estimated for low-risk and high-risk families, and for English-speaking and language minority families.

## Results

Table 1 presents descriptive statistics for key predictors, mediators, moderators, and outcome variables.

### Research Questions 1a and 1b: The Total Effects of Birth Order Moderated by Cumulative Risks and Language Minority Index

We first examined the overall effect of birth order on children's developmental outcomes. In the whole sample, firstborn and later-born children did not differ in their cognitive skills at 24 months [*b* (*SE*) = −0.62 (0.39), 95% CI = (−1.40, 0.14), *p* = 0.112; see Table 2]. Standardizing the coefficient yielded a 0.06 SD difference in cognitive skills between firstborn and later-born children.

**Table 2:** Unconditional and conditional total, indirect, and direct effects of birth order on children's cognitive and school readiness skills.

	**Cognitive skills at 24 mon**							**Literacy skills at preschool**							**Math skills at preschool**						
	**Coef**	**(SE)**		**LL CI^c^**	**UL CI^c^**	**Chi^**2**^**		**Coef**	**(SE)**		**LL CI**	**UL CI**	**Chi^**2**^**		**Coef**	**(SE)**		**LL CI**	**UL CI**	**Chi^**2**^**
**Total effects**															
Whole	−0.62	(0.39)		−1.40	0.14			−1.34	(0.35)^***^		−2.03	−0.65			−0.66	(0.32)^*^		−1.29	−0.03		
sample
CR^a^ = 0	−1.05	(0.47)^*^		−1.97	−0.14	3.54^∧^		−1.99	(0.44)^***^		−2.86	−1.12	10.00^**^		−1.01	(0.38)^**^		−1.76	−0.26	3.13^∧^	
CR = 3	0.54	(0.71)		−0.85	1.92			0.45	(0.60)		−0.72	1.63			0.31	(0.63)		−0.92	1.54		
LMI^b^ = 0	−1.14	(0.41)^**^		−1.94	−0.33	18.08^***^		−0.30	(0.06)^***^		−0.42	−0.19	4.35^*^		−0.88	(0.34)^**^		−1.55	−0.22	4.39^*^	
LMI = 3	2.56	(0.84)^**^		0.92	4.19			1.48	(0.85)^∧^		−0.19	3.14			1.04	(0.87)		−0.67	2.74		
**Indirect effects via home learning activities**															
Whole	−0.32	(0.07)^***^		−0.46	−0.18			−0.27	(0.05)^***^		−0.38	−0.17			−0.19	(0.04)^***^		−0.28	−0.11		
sample
CR = 0	−0.43	(0.08)^***^		−0.58	−0.28	7.67^**^		−0.29	(0.06)^***^		−0.41	−0.17	0.35		−0.21	(0.05)^***^		−0.30	−0.11	0.36	
CR = 3	−0.02	(0.13)		−0.27	0.24			−0.23	(0.10)^*^		−0.42	−0.03			−0.16	(0.07)^*^		−0.30	−0.02		
LMI = 0	−0.38	(0.07)^***^		−0.53	−0.24	9.67^**^		−0.30	(0.06)^***^		−0.42	−0.19	2.65		−0.21	(0.05)^***^		−0.31	−0.12	2.56	
LMI = 3	0.12	(0.15)		−0.18	0.42			−0.09	(0.12)		−0.33	.14			−0.07	(0.09)		−0.23	0.10		
**Direct effects**															
Whole	−0.30	(0.39)		−1.06	0.45			−1.07	(0.35)^***^		−1.76	−0.38			−0.47	(0.32)		−1.10	0.17		
sample
CR = 0	−0.62	(0.46)		−1.53	0.28	1.99		−1.70	(0.44)^***^		−2.57	−0.83	9.64^**^		−0.80	(0.38)^*^		−1.56	−0.05	2.95^∧^	
CR = 3	0.55	(0.70)		−0.81	1.92			0.68	(0.60)		−0.49	1.85			0.47	(0.62)		−0.75	1.69		
LMI = 0	−0.75	(0.41)^∧^		−1.55	0.04	13.41^***^		−1.40	(0.37)^***^		−2.13	−0.66	10.82^**^		−0.67	(0.34)^*^		−1.33	0.00	3.78^∧^	
LMI = 3	2.44	(0.84)^**^		0.80	4.08			1.57	(0.84)^∧^		−0.09	3.22			1.10	(0.86)		−0.59	2.80		

a *CR refers to cumulative risks*.

b *LMI refers to language minority index*.

c *LL CI and UL CI refer to lower and upper limits of 95% confidence interval*.

*All models controlled for cumulative risks, language minority index, children's sex, age of assessment, birth weight, multiple birth status, race, mothers' age at child birth, number of siblings, total number of household members, mothers' working status, region of residence, degree of urbanicity, the number of hours per week children spent in non-parent childcare arrangement, and mothers' self-reported health*.

*** *p < 0.001*;

** *p < 0.01*;

* *p < 0.05*;

∧ *p < 0.10*.

*Source: U.S. Department of Education, National Center for Education Statistics, Early Childhood Longitudinal Study, Birth Cohort (ECLS-B), 24 month and preschool data*.

However, the total effect of birth order was marginally moderated by cumulative risks [*b* (*SE*) = 0.53 (0.28), 95% CI = (−0.02, 1.08), *p* = 0.061; see Model 1a, Table 3] and significantly moderated by language minority index [*b* (*SE*) = 1.23 (0.29), 95% CI = (0.67, 1.80), *p* < 0.001; see Model 2a, Table 3]. In low-risk or English-speaking families, firstborns showed a 0.10 SD advantage in cognitive skills over later-born children (see Table 2). In contrast, there was no group difference in high-risk families, and a later-born advantage (0.23 SD) in language minority families.

**Table 3:** Cumulative risk and language minority index moderated the effects of birth order on developmental outcomes via home learning activities.

		**24 months: cognitive skills**		**4 years: literacy skills**		**4 years: math skills**	
		**Coef**.	**(SE)**	**Coef**.	**(SE)**	**Coef**.	**(SE)**
		**Model 1a**		**Model 1b**		**Model 1c**	
Moderating path c	Later-born	−1.06	(0.47)^*^	−2.01	(0.44)^***^	−1.03	(0.38)^**^
	CR^a^	−1.57	(0.23)^***^	−2.59	(0.22)^***^	−2.43	(0.21)^***^
	Later-born × CR	0.53	(0.28)^∧^	0.80	(0.26)^**^	.43	(0.25)^∧^
		**Model 2a**		**Model 2b**		**Model 2c**	
	Later-born	−1.14	(0.41)^**^	−1.71	(0.37)^***^	−0.89	(0.34)^**^
	LMI^b^	−2.38	(0.27)^***^	−1.68	(0.27)^***^	−0.84	(0.28)^**^
	Later-born × LMI	1.23	(0.29)^***^	1.02	(0.30)^**^	0.61	(0.31)^*^
		**Model 3a**		**Model 3b**		**Model 3c**	
Moderating path c'	Later-born	−0.62	(0.46)	−1.70	(0.44)^***^	−0.80	(0.38)^*^
	CR	−1.25	(0.23)^***^	−2.42	(0.22)^***^	−2.31	(0.21)^***^
	Later-born × CR	0.39	(0.28)	0.79	(0.26)^**^	0.42	(0.25)^∧^
		**Model 4a**		**Model 4b**		**Model 4c**	
	Later-born	−0.75	(0.41)^∧^	−1.40	(0.37)^***^	−0.67	(0.34)^*^
	LMI	−2.00	(0.28)^***^	−1.47	(0.27)^***^	−0.69	(0.27)^*^
	Later-born × LMI	1.06	(0.29)^***^	0.99	(0.30)^**^	0.59	(0.30)^∧^
							
		**24 months: learning activities**		**4 years: learning activities**		
		**Coef**.	**(SE)**	**Coef**.	**(SE)**		
		**Model 5a**		**Model 5c**			
Moderating path a	Later-born	−0.52	(0.08)^***^	−0.47	(0.08)^***^		
	CR	−0.38	(0.05)^***^	−0.29	(0.05)^***^		
	Later-born × CR	0.17	(0.06)^**^	0.04	(0.06)		
		**Model 6a**		**Model 6c**			
	Later-born	−0.47	(0.08)^***^	−0.49	(0.08)^***^		
	LMI	−0.45	(0.06)^***^	−0.40	(0.06)^***^		
	Later-born × LMI	0.20	(0.06)^**^	0.11	(0.07)^∧^	

a *CR refers to cumulative risks*.

b *LMI refers to language minority index*.

*All models controlled for cumulative risks, language minority index, children's sex, age of assessment, birth weight, multiple birth status, race, mothers' age at child birth, number of siblings, total number of household members, mothers' working status, region of residence, degree of urbanicity, the number of hours per week children spent in non-parent childcare arrangement, and mothers' self-reported health*.

*** *p < 0.001*;

** *p < 0.01*;

* *p < 0.05*;

∧ *p < 0.10*.

*Source: U.S. Department of Education, National Center for Education Statistics, Early Childhood Longitudinal Study, Birth Cohort (ECLS-B), 24 month and preschool data*.

At preschool (48 months), there was a firstborn advantage in children's literacy [*b* (*SE*) = −1.34 (0.35), 95% CI = (−2.03, −0.65), *p* < 0.001] and math [*b* (*SE*) = −0.66 (0.32), 95% CI = (−1.29, −0.03), *p* = 0.040; see Table 2] skills in the whole sample. Firstborn children scored 0.13 SD and 0.07 SD higher in literacy and math assessments than later-born children.

Notably, the total effect of birth order on literacy skills was moderated by both cumulative risks [*b* (*SE*) = 0.80 (0.26), 95% CI = (0.30, 1.31), *p* = 0.002; see Model 1b, Table 3] and language minority index [*b* (*SE*) = 1.02 (0.30), 95% CI = (0.43, 1.61), *p* = 0.001; see Model 2b, Table 3]. Likewise, the total effect of birth order on math skills was marginally moderated by cumulative risks [*b* (*SE*) = 0.43 (0.25), 95% CI = (−0.06, 0.92), *p* = 0.085; see Model 1c, Table 3] and significantly moderated by language minority index [*b* (*SE*) = 0.61 (0.31), 95% CI = (0.01, 1.21), *p* = 0.046; see Model 2c, Table 3]. In low-risk or English-speaking families, firstborns showed a 0.16–0.19 SD advantage in literacy skills and a 0.09–0.10 SD advantage in math skills. However, in high-risk families or language minority families, later-born children showed equivalent or marginally (*p* = 0.082) better skills compared to firstborns (see Table 2).

### Research Questions 2a and 2b: The Mediating Role of Home Learning Environment and the Moderated Mediation Models

We next examined whether children's engagement in home learning activities mediated the effect of birth order on child outcomes (see Figure 1B). These mediation analyses were followed by moderated mediation models, in which we tested whether cumulative risks and language minority index moderated the direct and indirect effects of birth order (see Figure 1C).

#### Indirect Effects via Home Learning Activities

As shown in Table 2, children's engagement in home learning activities mediated the effect of birth order on children's cognitive skills at 24 months in the whole sample [Unconditional indirect effect: *b* (*SE*) = −0.32 (0.07), 95% CI = (−0.46, −0.18), *p* < 0.001]. Compared to later-born children, firstborn children engaged in home learning activities more frequently, which in turn related to higher levels of cognitive skills.

Notably, the association between birth order and children's engagement in home learning activities at 24 months was moderated by both cumulative risks [*b* (*SE*) = 0.17 (0.06), 95% CI = (0.05, 0.28), *p* = 0.005; see Model 5a, Table 3] and language minority index [*b* (*SE*) = 0.20 (0.06), 95% CI = (0.08, 0.33), *p* = 0.001; see Model 6a, Table 3]. Specifically, the conditional indirect effect favored firstborns for children from low-risk or English-speaking families but was non-significant for children from high-risk or language minority families (see Table 2).

At preschool, the unconditional indirect effect via learning activities was significant for both literacy skills [*b* (*SE*) = −0.27 (0.05), 95% CI = (−0.38, −0.17), *p* < 0.001] and math skills [*b* (*SE*) = −0.19 (0.04), 95% CI = (−0.28, −0.11), *p* < 0.001; see Table 2]. Compared to later-born children, firstborn children engaged in home learning activities more frequently, which in turn predicted higher levels of literacy and math skills.

Cumulative risks [*b* (*SE*) = 0.04 (0.06), 95% CI = (−0.08, 0.15), *p* = 0.552; see Model 5c, Table 3] did not significantly moderated this indirect effect. Although language minority index marginally moderated the indirect effect [*b* (*SE*) = 0.11 (0.07), 95% CI = (−0.02, 0.25), *p* = 0.098; see Model 6c, Table 3], the differences in the magnitude of indirect effect between English-speaking and language minority families did not reach the level of significance (see Chi^2^ tests in Table 2).

#### Direct Effects of Birth Order

At 24 months, the unconditional direct effect of birth order on cognitive skills was non-significant [*b* (*SE*) = −0.30 (0.39), 95% CI = (−1.06, 0.45), *p* = 0.432; see Table 2]. The direct effect did not vary by cumulative risks [*b* (*SE*) = 0.39 (0.28), 95% CI = (−0.15, 0.94), *p* = 0.158; see Model 3a, Table 3], but varied by language minority index [*b* (*SE*) = 1.06 (0.29), 95% CI = (0.49, 1.63), *p* < 0.001; see Model 3a, Table 3]. The conditional direct effect indicated a marginal, firstborn advantage (*p* = 0.064) in English-speaking families, but a later-born advantage in language minority families (see Table 2).

At preschool, birth order had a significant, unconditional direct effect on literacy skills [*b* (*SE*) = −1.07 (0.35), 95% CI = (−1.76, −0.38), *p* = 0.002], but a non-significant effect on math skills [*b* (*SE*) = −0.47 (0.32), 95% CI = (−1.10, 0.17), *p* = 0.148; see Table 2]. These direct effects were also moderated or marginally moderated by cumulative risks [Literacy: *b* (*SE*) = 0.79 (0.26), 95% CI = (0.29, 1.30), *p* = 0.002; see Model 3b, Table 3; Math: *b* (*SE*) = 0.42 (0.25), 95% CI = (−0.06, 0.91), *p* = 0.086; see Model 3c, Table 3] and language minority index [Literacy: *b* (*SE*) = 0.99 (0.30), 95% CI = (0.40, 1.58), *p* = 0.001; see Model 4b, Table 3; Math: *b* (*SE*) = 0.59 (0.30), 95% CI = (0.00, 1.18), *p* = 0.052; see Model 4c, Table 3]. As shown in Table 2, the conditional direct effects on literacy and math skills favored firstborns for children from low-risk or English-speaking families but were non-significant or marginally favoring later-born children (*p* = 0.063 on literacy skills in language minority families) for those from high-risk or language minority families.

## Discussion

Existing theories on the role of birth order in child development have revealed the complicated influences of family resources distribution, family dynamics, and socioecological context. Although prior work has documented a firstborn advantage in children's development of early cognitive and school readiness skills (e.g., Barreto et al., 2017; Lehmann et al., 2018), how and in which context(s) birth order plays a role are not yet fully understood. The current study examined the extent to which the effect of birth order was mediated by home learning environment and moderated by children's exposure to cumulative risks and language minority experiences.

Three key findings have emerged. First, the firstborn advantage in early cognitive and school readiness outcomes was primarily observed in children from low-risk or English-speaking families. However, in high-risk or language minority families, later-born children showed similar or even higher levels of skills than firstborns. Second, children's engagement in home learning activities mediated the effect of birth order. However, the direction and magnitude of the indirect effects varied by cumulative risks and language minority index. Finally, in low-risk or English-speaking families, birth order had a direct effect favoring firstborns on child outcomes, above and beyond children's engagement in home learning activities. However, the direct effect favored later-borns or was non-significant in high-risk or language minority families. Together, these findings highlight the context-specificity of the effect of birth order and reveal the complex interactions among a family's sibling structure, resource distributions, and ecological context.

### The Overall Effects of Birth Order Varied by Cumulative Risks and Language Minority Index

In line with the bioecological model, which emphasizes the contextual influences on family dynamics (Bronfenbrenner and Morris, 2006), our findings suggested that the firstborn advantage was more evident in low-risk or English-speaking families than in high-risk or language minority families. The magnitude of the firstborn advantage on child outcomes, especially children's literacy skills at preschool, became smaller as cumulative risks increased and diminished to non-significant for children exposed to three or more of the following risk factors: poverty, single-mother household, low maternal education, and maternal depression. Consistent with previous studies that showed a smaller or non-significant firstborn advantage in language and school outcomes for children from lower-SES families (Zambrana et al., 2012; Cheng et al., 2013; Kim et al., 2018), these findings offered new evidence to the limited work on the birth order effect in families experiencing multiple risk factors, and challenged the universality of the classic birth order theories (Blake, 1981; Zajonc, 1983).

Additionally, the current study was the first one showing that the effect of birth order varied by children's home language environment. While there was a firstborn advantage in English-speaking families, in language minority families, being later-born *positively* predicted children's cognitive skills at 24 months and marginally predicted literacy skills at preschool. Previous work has shown a smaller or non-significant firstborn advantage for children from ethnic minority (Lehmann et al., 2018) and certain immigrant groups (Isungset et al., 2020). Consistent with the social learning theories (Bandura, 1977), our findings further highlighted the contributions of older siblings to their younger siblings' early cognitive and language development in language minority families (Hurtado-Ortiz and Gauvain, 2007; Obied, 2009; Kibler et al., 2016).

### The Mediating Role of Home Learning Activities Varied by Cumulative Risks and Language Minority Index

According to the resource dilution and confluence theories, the effect of birth order on child development can be explained by firstborn children's greater access to family resources and high-quality learning environment at home (Blake, 1981; Zajonc, 1983). In line with these theoretical hypotheses, in low-risk or English-speaking families, children's engagement in home learning activities mediated the effect of birth order on children's cognitive skills at 24 months and literacy and math skills at preschool. Compared to firstborn children, later-born children engaged in learning activities less frequently, which in turn predicted less advanced cognitive and school readiness. Consistent with previous literature (Raikes et al., 2006; Peyre et al., 2016), these findings confirmed a dilution of parents' interpersonal investments for later-born children in low-risk or English-speaking families.

However, the mediation pathway via home learning activities did not apply to children from high-risk or language minority families at 24 months. For these children, birth order was not associated with their engagement in learning activities. When parents have limited ability to engage their children in learning activities due to challenges such as low educational level, language barriers, or maternal depression, there may not be obvious advantages of being firstborn (Downey, 2001; Cheng et al., 2013). Moreover, older siblings in these families might step into their parents' shoes and engage their younger siblings in learning activities, which compensates the lack of parental support (Gregory, 2001; Zentella, 2005; Kibler et al., 2016; Luo and Tamis-LeMonda, 2019). Furthermore, parents from high-risk or language minority families tend to be less familiar with the school system and have less knowledge of child development than their counterparts in more affluent or English-speaking families (Keels, 2009; Rowe et al., 2016; Suskind et al., 2018). Older siblings' school experiences might help these parents recognize the importance of early literacy development (Sawyer et al., 2018) and motivate parents to facilitate more learning activities early on. These positive impacts of older siblings in high-risk or language minority families might be especially strong during toddlerhood when children spend most of their time at home.

### The Direct Effect of Birth Order Beyond Home Learning Environment

In low-risk or English-speaking families, firstborn children showed better literacy and math skills at preschool than later-born children, after controlling for children's engagement in home learning activities. These findings suggested that birth order might affect child development in ways beyond home learning activities. Perhaps later-born children experienced dilution in other aspects of their home learning environment, such as quality time with parents (Price, 2008) and parental language input (Hoff, 2006). Alternatively, there is evidence that parents tend to have lower academic expectations for their later-born children (Kim, 2020) and use less strict disciplinary strategies with their later-born children (Hotz and Pantano, 2015), both of which may have been related to lower developmental outcomes of the later-born children.

In high-risk or language minority families, however, being later-born had a non-significant or even positive direct effect on child outcomes. The social learning theories (Bandura, 1977) propose various mechanisms through which older siblings facilitate children's learning experiences, including intimate sibling relationships, socialization processes, and positive sibling exchanges (Whiteman et al., 2011; Howe et al., 2014). Indeed, children might engage in informal learning activities with their older siblings that were not captured in the current study, such as free play, daily conversations, and drawing (Kibler et al., 2016). The supportive role of older siblings may be more salient in high-risk or language minority families where parents face financial, cultural, or language obstacles to offering rich home learning environment for their children. In language minority families, older siblings can be more acculturated and have better English skills than their parents and may therefore provide quality support to their younger siblings' language and literacy skills in English (Farver et al., 2013).

### Limitations and Future Directions

The current study has several limitations. We only considered children's firstborn vs. later-born status, without examining the total number of older/younger siblings, their gender, and the age difference between them and the target child, all of which might influence the direction and magnitude of the birth order effect. For example, siblings with small or large age gaps may interact with and learn from each other in different ways. Additionally, we did not have any data on language minority children's school readiness skills in their home language, and those children (1.37%) with insufficient English fluency at preschool were excluded from the analyses. Future research should explore whether birth order shows differential effects on children's school readiness skills in English and in the home language. Moreover, we only examined children's engagement in book-reading, storytelling, and singing activities. There are many more aspects of home learning environment, such as other learning activities (e.g., toy play and drawing), the availability of literacy materials, and language use by family members, which may further elucidate the effect of birth order on children's developmental outcomes (Bridges and Hoff, 2014). Finally, the (moderated) mediation models were based on cross-sectional data, which preclude any conclusion on causality. The relations between home learning environment and child developmental outcomes are likely bidirectional, such that children with more advanced cognitive and school readiness skills might actively engage in more learning activities with their family members (Tamis-LeMonda et al., 2019). Longitudinal work is needed to understand the directionality of these associations.

## Conclusions

Findings of this study reveal the complex associations among sibship structure, home learning environment, and early childhood development. The effect of birth order showed strong context-specificity. Unlike low-risk or English-speaking families, where there was a firstborn advantage in child outcomes, there was no difference between firstborn and later-born or even a later-born advantage in high-risk or language minority families. The direct effect of birth order on child outcomes and its indirect effects via home learning activities varied by cumulative risks and language minority index. These findings underscore the need for integrating classic birth order theories, social learning theories, and ecological systems approach to interpret differential birth order effects in diverse contexts. Practically, early preventions and interventions may support children from high-risk or language minority families by encouraging sibling interactions to amplify the positive role older siblings play in child development.

## Data Availability Statement

The data analyzed in this study was obtained from the Early Childhood Longitudinal Study–Birth Cohort study. Access to the restricted-use datasets can be requested at: https://nces.ed.gov/ecls/birthdatainformation.asp.

## Ethics Statement

Ethical review and approval was not required for the study on human participants in accordance with the local legislation and institutional requirements. Written informed consent to participate in this study was provided by the participants' legal guardian/next of kin.

## Author Contributions

RL and LS contributed to conceptualization. RL and I-MC contributed in data analysis. RL involved manuscript writing. LS and I-MC reviewed and edited the manuscript. All authors contributed to the article and approved the submitted version.

## Conflict of Interest

The authors declare that the research was conducted in the absence of any commercial or financial relationships that could be construed as a potential conflict of interest.

## Publisher's Note

All claims expressed in this article are solely those of the authors and do not necessarily represent those of their affiliated organizations, or those of the publisher, the editors and the reviewers. Any product that may be evaluated in this article, or claim that may be made by its manufacturer, is not guaranteed or endorsed by the publisher.
